# Applying the disability-adjusted life year to track health impact of social franchise programs in low- and middle-income countries

**DOI:** 10.1186/1471-2458-13-S2-S4

**Published:** 2013-06-17

**Authors:** Dominic Montagu, Lek Ngamkitpaiboon, Susan Duvall, Amy Ratcliffe

**Affiliations:** 1 Global Health Group, University of California San Francisco, CA, USA; 2 Population Services International, Washington DC, USA; 3 Independent global health consultant, Seattle, WA, USA

## Abstract

**Background:**

Developing effective methods for measuring the health impact of social franchising programs is vital for demonstrating the value of this innovative service delivery model, particularly given its rapid expansion worldwide. Currently, these programs define success through patient volume and number of outlets, widely acknowledged as poor reflections of true program impact. An existing metric, the disability-adjusted life years averted (DALYs averted), offers promise as a measure of projected impact. Country-specific and service-specific, DALYs averted enables impact comparisons between programs operating in different contexts. This study explores the use of DALYs averted as a social franchise performance metric.

**Methods:**

Using data collected by the Social Franchising Compendia in 2010 and 2011, we compared franchise performance, analyzing by region and program area. Coefficients produced by Population Services International converted each franchise's service delivery data into DALYs averted. For the 32 networks with two years of data corresponding to these metrics, a paired t-test compared all metrics. Finally, to test data reporting quality, we compared services provided to patient volume.

**Results:**

Social franchising programs grew considerably from 2010 to 2011, measured by services provided (215%), patient volume (31%), and impact (couple-years of protection (CYPs): 86% and DALYs averted: 519%), but not by the total number of outlets. Non-family planning services increased by 857%, with diversification centered in Asia and Africa. However, paired t-test comparisons showed no significant increase within the networks, whether categorized as family planning or non-family planning. The ratio of services provided to patient visits yielded considerable range, with one network reporting a ratio of 16,000:1.

**Conclusion:**

In theory, the DALYs averted metric is a more robust and comprehensive metric for social franchising than current program measures. As social franchising spreads beyond family planning, having a metric that captures the impact of a range of diverse services and allows comparisons will be increasingly important. However, standardizing reporting will be essential to make such comparisons useful. While not widespread, errors in self-reported data appear to have included social marketing distribution data in social franchising reporting, requiring clearer data collection and reporting guidelines. Differences noted above must be interpreted cautiously as a result.

## Background

Private providers outnumber public providers in nearly all low- and middle-income countries (LMICs), delivering more than 50% of all healthcare services in Africa and Asia and more than 70% of all healthcare in the most populous countries in these regions: Nigeria, India, Pakistan, Bangladesh, and Indonesia [1]. Given private health facilities' large share of national health markets, LMIC national health systems are increasingly challenged to ensure these private providers sufficiently advance public health goals and adhere to national standards of care.

Social franchising is an innovative way of leveraging existing private sector infrastructure in LMICs to serve these national public health goals. To do so, social franchising promotes and improves clinical services among existing private providers through technical assistance and the application of social marketing techniques, a health intervention that has proven successful in fostering widespread access to and use of public health commodities in LMIC countries [2,3]. As the fastest growing mechanism for engaging private practitioners in national public health initiatives, this social franchising approach is supported by a highly collaborative community of practice and a growing body of evidence that demonstrates social franchising's ability to improve service quality, increase access to essential services, and serve the poor [4,5].

Social franchises engage existing for-profit, private clinics in a contractual exchange. The franchise program, nearly always run by a non-governmental organization (NGO), builds providers' capacity in new clinical methods, clinic management, business skills, and marketing and advertising techniques. Doing so builds demand for the health services offered. The program also organizes ongoing education and technical support as well as access to subsidized commodities and medicines which are often unavailable. Service quality is further supported through franchise-mediated linkages to private and national referral systems. In exchange, providers who enroll as franchisees commit to providing the health services that the implementing organization prioritizes, often low-margin services such as family planning or antenatal care, or socially unattractive services such as treatment for tuberculosis (TB) or HIV/AIDS. Franchise providers also are required to adhere to clearly defined clinical, reporting, and pricing practices. Increased client load, the resulting increase in profits, training opportunities, and reputation enhancement are the most frequently cited motivations for providers to join a social franchise [6,7].

**Figure 1:**
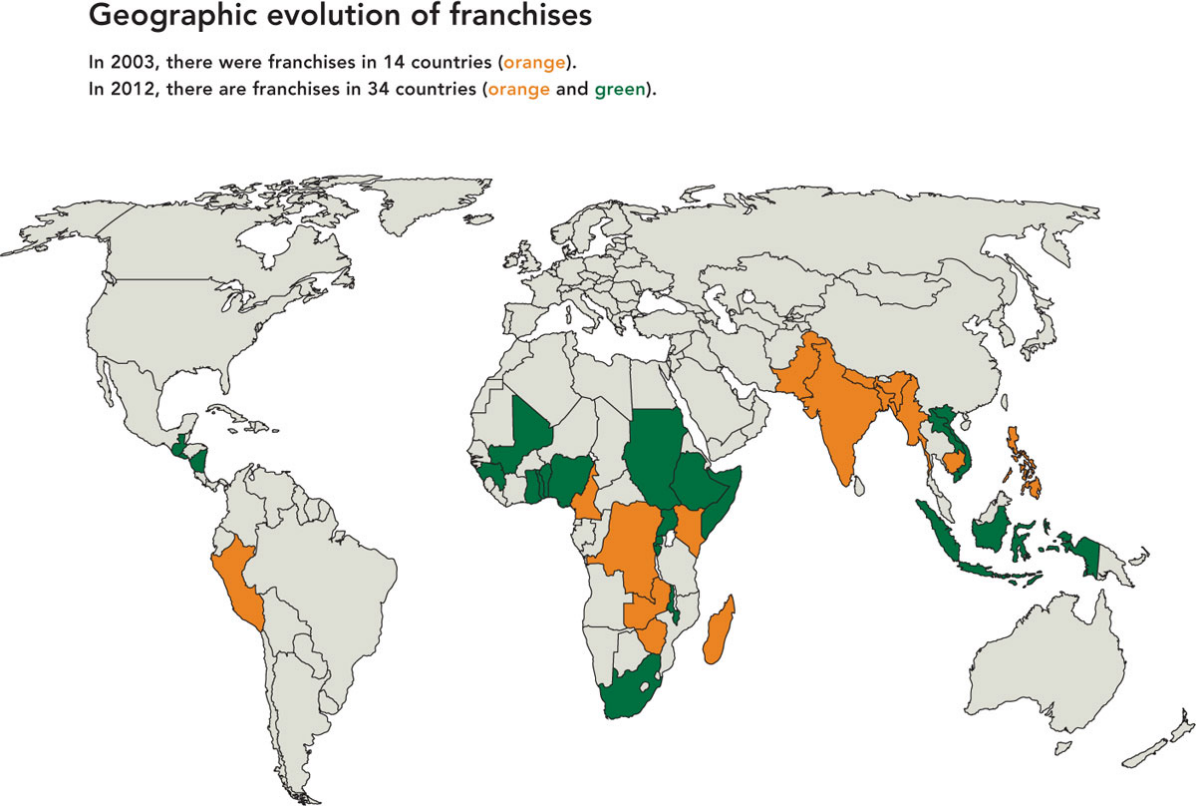
**Global growth of social franchising programs, 2003 versus 2012**.

Begun in the early 1990s in Pakistan and Nepal, social franchising programs have steadily expanded throughout the developing world; as of June 2012, more than 50 programs operate in 35 countries [8] (see map, Figure 1). Initially, participating clinics and providers offered only family planning services, but many franchise service portfolios have expanded in recent years to include pediatric and maternal care as well as infectious disease testing and treatment, among others. More than half of the world's social franchise programs now offer a range of non-family planning services, and the number of program areas addressed by each franchise is growing each year [8]. While social franchises are often linked to large-scale social marketing programs which promote and distribute non-clinical health commodities (e.g., condoms or mosquito bednets) through retail outlets, the franchises themselves are, by definition, focused on clinical service delivery. Currently, two large international NGOs, Population Services International (PSI) and Marie Stopes International (MSI), operate or support the majority of global social franchise programs, 25 and 10 programs respectively [9,10].

The growth in the number of programs has led to the development of a global community of practice among social franchisors. Coordinated by the University of California, San Francisco (UCSF)'s Global Health Group, this network of social franchising implementors facilitates information exchanges and supports almost all of the known social franchising programs, compiling data annually about its members in a *Compendium of Clinical Social Franchising Programs *[8,11]. The Social Franchising Compendia are comprised of both large and small franchise programs. Large programs are defined as reporting an operating budget of more than $500,000 per year and including more than 50 member clinics. From 2010-2011, the Compendia gathered data on 58 social franchise networks, spanning Asia, Africa, and Latin America.

Increased confidence in this service delivery model on the part of donor agencies and NGOs accounts, in part, for the rapid expansion of social franchising. These stakeholders believe that franchises increase access to important clinical services by extending the geographic reach of the government healthcare system. Related clinical service models, such as NGO delivery or one-time training programs, face challenges in providing widely distributed services. It can be difficult for these models to meet high quality standards at outlets while offering only a low volume of services to a small catchment area and patient base. In contrast, by leveraging existing infrastructure and *in situ *skilled providers, social franchise networks are theoretically able to assure quality at comparatively low cost, even in low volume settings [2].

As with any health intervention, understanding the health impact of social franchising programs is essential, to inform decision making and to demonstrate the viability and value of this innovative approach. Currently, programs track a number of service delivery measures: the number of patient visits, not tracked by individual patients, for any cause or service (called "patient volume" hereafter); the number of clinics operating (called "number of outlets" hereafter); and (rarely) the number of prevention or treatment interventions received by patients (called "services provided" hereafter). Family planning-focused programs frequently track and report CYPs, a measure of projected impact that can be applied to all family planning methods based on weights to approximate the number of years a method safeguards against pregnancy and the number of products or services provided. For example, 120 condoms are considered to protect the couple from pregnancy for one year, and are thus, equal to one CYP, as are 13 cycles of monthly contraceptive pills, or .26 of a 5-year implant. (Note that the average implant is estimated to provide only 3.8 CYPs, not five, because some 5-year implants are removed early; hence .26 implants leads to one CYP.) [12].

These process measures are widely acknowledged as crude and poorly correlated with the true health impact that these programs likely achieve [13]. Patient volume and number of outlets have inherent problems: the former includes no information on severity of disease, while the latter does not reflect information on the patients served or the types of illnesses and health conditions treated [14]. Although the CYP metric is a good aggregate measure of family planning program projected impact, it is a metric of protection, not health impact, and it is limited to one program area only. As social franchises increasingly expand their portfolio of services beyond family planning, CYPs will capture only a small portion of all care offered.

Program managers and donors currently seek more robust measures of program impact, both to benchmark program performance against appropriate comparators, and to track year-to-year improvement [15-18]. A standardized metric for projected health impact would enable these program comparisons and allow for more accurate assessments of social franchising program variations, after accounting for changes in population health and service delivery. Such a method would also provide a basis for more accurate analysis of the cost-effectiveness of complex delivery systems and programs.

An existing metric, DALYs averted, offers promise as an impact measure of social franchising programs. This metric is based on the DALY, the standard unit representing disease burden in a population, originally developed for the World Bank in 1990 and adopted by the World Health Organization (WHO) in 2000 as part of the Global Burden of Disease Study [19,20]. Disease burden can be expressed as the number of DALYs lost due to a health condition, either from premature death (mortality) or disability (morbidity), as compared to an ideal life expectancy [20]. The DALYs averted measure, then, denotes the disability-adjusted life years that are not lost - or, are averted - as a result of a health intervention. The attraction of using a metric based on the DALY is that the DALY incorporates both mortality and morbidity, it is widely used by global development agencies, it enables comparison across countries on an standardized scale, and it can be aggregated or disaggregated by disease [21,22].

The DALYs averted metric is intended conceptually to be the inverse of DALYs in burden and thus benefits from a growing understanding of what a DALY, as a unit, is. Such attributes allow decision makers to use DALYs for comparing the overall burden of disease due to TB, malaria, diabetes, or other illnesses. Conversely, decision makers can also compare projected DALYs averted for understanding the potential to reduce burden with specific interventions, and to allocate resources based on these data.

Despite its established importance in global burden of disease assessment, the DALY has only recently been adapted into a measure of the impact of healthcare interventions by service providers. Beginning in 2007, PSI initiated the use of an impact metric based on the DALY. In each of the 58 countries where PSI operated, researchers calculated a country- and disease-specific DALY coefficient for each prevention or treatment intervention [23]. These models project the impact of specific products and services across a wide range of program areas, such as male circumcision, malaria rapid diagnostic test kits, intrauterine device (IUD) insertions, and pneumonia treatment [24]. Note that PSI bases the DALYs averted metric on models of health impact which are necessarily different from the WHO and World Bank burden models of DALYs. PSI has also never used age weighting in DALY calculations.

PSI DALYs averted models are tailored to different diseases and interventions. As a generalized description, DALYs averted are calculated based on reduced risk in a population and the corresponding years of healthy life preserved. Using country-specific population and health data, the reduced risk of death is calculated by applying the demonstrated effectiveness of prevention or treatment to baseline disease burden. To calculate impact in DALYs averted, the reduction in mortality is multiplied by the number of years between average age at death for the targeted disease and ideal life expectancy. The ratio of years lost from death to years lost from disability is used to calculate the additional years that would be lost to disability for the same disease. The result of this calculation is a DALYs averted coefficient, the impact of a single product or service in years of healthy life preserved. Other publications describe the PSI methodology in more detail [24].

Each of PSI's DALYs averted models projects health impact from the use of one service or product delivered (e.g., a safe child delivery or the sale of a packet of oral rehydration salts (ORS) for pediatric diarrhea), producing a coefficient for this unit. Besides being product- or service-specific, this coefficient is also country-specific, as the number of DALYs averted by any treatment or service varies according to the national burden of disease. For example, in a country with a low malaria burden, the sale of a long-lasting, insecticide-treated net (LLIN) will have a small coefficient whereas this coefficient will be much larger in a country with higher incidence and widespread malaria. Therefore, the DALYs averted coefficient for an LLIN in Nicaragua will differ sharply from that used for an LLIN in Benin. To generate estimates of the number of DALYs averted by a specific service or product intervention, the coefficient is then applied to the total number of services or products distributed within the country during the past year. For example, the LLIN coefficient for Benin is multiplied by the number of LLINs distributed, resulting in the total number of DALYs averted by the LLIN intervention in Benin.

The final model outputs for each intervention, DALYs averted, can serve as a program management tool as these outputs allow comparisons of impact between countries and between different interventions offered within each country program. Organizations can use the DALYs averted metric to benchmark program impact and determine program emphasis and design, as described elsewhere [25]. Thus far, both PSI and MSI have adopted DALYs averted for internal program management use; other organizations are considering doing the same.

For managing social franchising programs, the DALYs averted metric would be useful for projecting program impact, by applying the coefficients to reported service delivery data, on a monthly, quarterly, or annual basis. DALYs averted can be considered for specific interventions within a health program area (e.g., HIV or family planning) for a social franchise network, country, or region. To understand the overall health impact of a social franchise program, the DALYs averted across all individual products or services can be summed. The resulting aggregate will show an estimate of the health impact of family planning services as well as the social franchising program's impact on malaria, diarrhea, and other targeted health conditions. With this aggregate, comparisons of overall effect between social franchising programs managed by different implementing organizations and operating in different national contexts are possible because DALYs averted adjusts for the burden of disease in each country.

### Study goals

This study seeks to demonstrate the application of a single, comprehensive health impact measure to social franchising programs. Our principal goal is to present the use of DALYs averted alongside currently used program output and impact measures - patient volume, number of outlets, number of services provided, and CYPs - to track progress across a global set of social franchising networks. We describe changes in social franchising impact over two years, 2010 to 2011, and highlight differences by region and program area. We also review the strengths and limitations of each of the individual metrics currently used by social franchising programs. In doing so, we discuss opportunities to incorporate DALYs averted as a social franchising metric, reviewing the programmatic decisions that could be informed by this metric. We hope this study will provide insight into the benefits and challenges of establishing a standardized impact measurement system for service delivery programs run by multiple implementing organizations.

## Methods

Data for this analysis comes from the 2010 and 2011 self-reported programmatic and services data collected by the Social Franchising Compendia for each year [8,11]. All social franchising programs known to UCSF's Global Health Group at the end of each year are included in the Social Franchising Compendia. To maximize the likelihood that all active social franchises are captured, those programs tracked by the Social Franchising Compendia are compared on a quarterly basis with other lists of social franchises (e.g., those compiled by the Center for Health Market Innovation and the International Centre for Social Franchising), in addition to conducting a thorough search of programs referenced on the internet, and canvassing donors and implementers.

Nearly all Social Franchising Compendia programs voluntarily report service delivery data annually between February and April, using either internet-based survey forms or Adobe Portable Document Format (PDF) documents filled in by hand. Data are checked for empty response cells and for consistency with program descriptions. Programs report the total number of patient visits (patient volume) and the treatments, procedures, and products provided (services provided) for a wide range of conditions and diseases. In addition, programs report a range of aggregate program data, including the number of clinics included in the social franchising network, the number and type of providers per clinic, and the size of annual operating budgets. Additional file 1 provides an overview of the social franchising programs that are Compendia members.

The key output measures emphasized in this study - services provided, patient volume, and number of outlets - were directly reported by the social franchising networks to the Social Franchising Compendium. These annual service delivery data formed the basis for calculating both DALYs averted and CYPs. CYPs were calculated using the 2011 USAID conversion factors specific to each family planning method, called CYP coefficients in this paper [12].

To apply DALYs averted to franchised program services, we adopted the most widely used tools for estimating the number of DALYs averted from service delivery data that we are aware of, the health impact estimation models used by PSI. PSI's DALYs averted models are appealing for this analysis because of their breadth: these models provide an estimate of per-unit impact of 44 different service and product interventions, many of which are offered by social franchisors and used in the LMICs where most social franchising programs are currently located. Therefore, the country- and service-specific DALYs averted coefficients produced by these models can be easily applied to multiple social franchises, regardless of their program affiliation.

To estimate DALYs averted, we multiplied the appropriate PSI DALYs averted country- and service-specific coefficients to the service delivery data reported by the social franchising program of interest. For example, the Bangladesh DALYs averted coefficients were used for determining the number of DALYs averted by each of the services offered by the Blue Star Bangladesh social franchise. Once DALYs averted were calculated for each service reported, we totalled all of them to produce an aggregated estimate of the number of DALYs averted for that social franchising program in either 2010 or 2011.

Among the full dataset reported to the Social Franchising Compendium, certain services could not be paired with coefficients for estimating DALYs averted because of one of the following reasons: PSI did not have a DALYs averted model for the intervention; the PSI model did not include country data for the country where the intervention was reported; or the social franchise program reported insufficient information, prohibiting the pairing of the service with a DALYs averted coefficient. Additional file 2 presents the full set of interventions reported by social franchising networks. Note that most, but not all, of these interventions could be matched to a country- and intervention-specific coefficient to estimate DALYs averted. All of the family planning methods offered by social franchisors could be converted to CYPs.

A total of 58 social franchising networks reported data to the Social Franchising Compendium for the years 2010 and 2011, with 50 programs reporting data in 2010 [11] and 51 programs doing so in 2011 [8]. Spanning three continents and 36 countries, these social franchising networks vary in age and size, with Asian networks being the oldest and largest. The oldest network is Sangini Franchising, launched in Nepal in 1994, while the youngest five networks were launched in 2011 in Laos, Mozambique, Sudan, Nicaragua, and Somaliland. Sub-Saharan Africa has the largest number of social franchising programs whereas Latin America has the fewest.

Fifty-one programs provided sufficient service delivery data to enable the calculation of DALYs averted or CYPs provided for 2010 or 2011. For the statistical analysis, we included only those programs from this sample which provided data for both years (N = 32). Half of these (16 programs) are controlled by or affiliated with PSI. The remaining half are either affiliated with MSI (6 programs), the US-based NGO, DKT International (1 program), or are independent (9 programs), meaning they are unaffiliated with an international NGO but may be linked to a local NGO or private organization within the country. While most of the non-PSI programs deliver healthcare in the same countries where PSI works, social franchising programs are also included from five countries where PSI is not present: Bangladesh, Indonesia, Peru, the Philippines, and Sierra Leone.

### Data adjustments

Certain non-PSI program data required some data extrapolation before we could derive the number of DALYs averted based on PSI's coefficients. For the five countries where PSI does not currently work (and therefore, where PSI has not yet determined country-specific coefficients for each health intervention), we needed to infer the value of the DALYs averted coefficients for that country. To do so, we applied the average of the intervention coefficient from countries in the same WHO region with a per-capita gross domestic product (GDP) within 300% of the non-PSI country. For example, to determine the Philippines coefficients, we used an average of those developed by PSI for Vietnam, Laos, and Cambodia. Once we identified the coefficients for each of these countries, we followed the process described above for calculating DALYs averted for the social franchising programs in these five countries.

We also needed to adjust non-PSI program data to fit the parameters used in PSI's DALYs averted models when the reported data relied on different treatment indicators or different services than those modelled. To make these adjustments, we first compared the products and services currently covered by PSI's DALYs averted models to those dispensed by non-PSI social franchises. Nine of the non-PSI programs reported the number of TB cases initiated on treatment rather than the number of TB cases completing treatment, a key distinction in the parameter used by the PSI TB DALYs averted model. Published correlations between reported outcomes (e.g., initiation of TB treatment) and outcomes needed for the DALYs averted model (e.g., treatment completion) guided the extrapolation of estimates for the non-PSI programs. We estimated completion rates using an average of 89.6% of TB treatment initiations, based on a WHO-led meta-analysis of public-private TB initiatives [26].

In one instance, we adjusted data by excluding data in one year because the data did not match the criteria required by the models. The PSI HIV Counseling and Testing (HCT) DALYs averted model for HIV counseling and testing distinguishes between six categories of patients receiving HIV voluntary counseling and testing services: HIV-negative and single; HIV-positive and single; HIV-negative in a concordant couple; HIV-positive in a concordant couple; HIV-negative in a discordant couple; and HIV-positive in a discordant couple. As non-PSI programs did not collect data on partner status in 2010, our analysis excluded their reported HCT data. We included these data from 2011, as a more detailed survey instrument recorded partner status from HCT client interactions.

A final set of adjustments concerned those non-PSI social franchising programs which dispensed products or services that are not currently supported by PSI's DALYs averted models. Examples of such services include labor and delivery, pediatric consultations, menstrual regulation services, and vaccinations. These products and services were not included in our analysis.

### Comparisons across years and regions

Social franchise networks were analyzed by size based on the number of outlets that were reported for each year. We used ANOVA to compare the average size of franchises across the regions.

We compared the sum of each of the metrics - services provided, DALYs averted, CYPs, and patient volume - for 2010 and 2011 for all of the social franchise programs reporting in each year and by region. We report the percent change from 2010 to 2011 for each of these metrics, including the changes by region. For statistical analysis, we used a paired t-test to detect differences in the metrics as reported across the two years by each of the social franchising networks. This test was intended to identify a common shift toward either increased or decreased output among those networks reporting data in both 2010 and 2011 (N = 32) for each metric. To understand the overall percent change for family planning and non-family planning services, we isolated services related to family planning from the programs reporting these data in both years (*n *= 30), summing up each of the metrics and considering the percent change over the two years. For statistical analysis, we again used a paired t-test to identify common differences year to year. We followed the same procedure for analyzing non-family planning services. Nineteen franchises were compared in the paired t-test.

In addition, we aggregated DALYs averted within program areas by region. All services were categorized into one of the following major program areas: family planning, sexual and reproductive health, maternal and child health, HIV/AIDS, malaria, diarrhea, acute respiratory disease, and tuberculosis.

To check reporting quality, we compared services provided to patient volume using a Pearson's correlation coefficient. Certainly, a weak relationship between services provided and the number of patient visits may not always indicate poor reporting. The relationship between these two metrics could be weak if different franchise networks offer different numbers of integrated services per patient. Moreover, it is worth noting that the unit of service may vary across interventions. For example, a year's supply of oral contraceptives would be counted as 13 services whereas one IUD would be counted as a single service, even though it is expected to protect against unintended pregnancy for longer than a year. Type of service, therefore, may affect the ratio between services provided and patient volume.

## Results

### Description of sample for analysis

Thirty-two franchise networks were represented in the set of services for which DALYs averted or CYPs provided could be calculated for the years 2010 and 2011. Networks included in the analysis represented 23 countries. Over the two years, these franchises reported 1,121,352,473 services provided through 22,941,811 patient visits. Additional file 3 shows a summary of reported data for all franchise networks in the analysis.

### Size of social franchising networks by region

Franchise networks in Asia tended to be larger than their counterparts in Latin America and Africa. On average, the number of outlets was different across the three regions for both years (ANOVA, p < .05). Asian social franchise programs had an average of 2,708 outlets across franchise networks in 2010. For the same year, Latin America had an average of 606 outlets and Africa had an average of 114 outlets. Number of outlets was not significantly different in 2011 compared to 2010 for any of the regions.

### Changes across the social franchising networks, 2010 to 2011

The years, 2010 to 2011, saw growth in social franchising across the set of networks, measured by output and impact. During this time, the services provided increased by 215% across the 32 franchise networks (Table 1). Asia and Africa saw considerable growth, 245% and 189% respectively, although African networks reported fewer than half the number of services provided overall compared to the Asian networks. For the 32 networks, the number of patient visits increased by 31% and CYPs rose by 86% over the two years. The number of DALYs averted grew by 519% across the full set of networks, with variation by region. African franchise networks reported the greatest change in DALYs averted, a 538% increase.

Latin America experienced a decline in services provided which also produced a decline in DALYs averted and CYPs. With just three countries (El Salvador, Guatemala, and Peru) offering small family planning programs, this region's social franchise programs are limited in size and scope, making its aggregate health impact results sensitive to shifts in individual programs, or program reporting. In this case, the exclusion of Guatemala's non-franchise condom distribution efforts, inappropriately included in 2010, reduced the number of DALYs averted in Latin America by two-thirds in 2011.

**Table 1:** Change in number of services provided, DALYs averted, CYPs, and patient volume for products and services eligible for analysis, 2010-11, by region

Region	2010	2011	% Change
**Asia **(*n *= 13)			

Services provided	178,386,749	615,368,041	245%

DALYs averted	865,187	5,306,229	513%

CYPs	5,749,247	7,934,530	38%

Patient volume	7,605,797	10,149,492	33%

**Africa **(*n *= 16)			

Services provided	81,368,197	235,315,665	189%

DALYs averted	691,776	4,411,879	538%

CYPs	892,064	4,693,329	426%

Patient volume	1,698,731	2,235,235	32%

**Latin America **(*n *= 3)			

Services provided	10,537,887	375,934	-96%

DALYs averted	14,770	3,645	-75%

CYPs	183,640	90,854	-54%

Patient volume	626,376	626,278	0%

**Total **(N = 32)			

Services provided	270,292,833	851,059,640	215%

DALYs averted	1,571,733	9,721,752	519%

CYPs	6,824,951	12,718,714	86%

Patient volume	9,930,806	13,011,005	31%

Over the two years, services in the franchise networks diversified; non-family planning services increased by 857% (Table 2). While these diverse types of interventions comprised 18% of the reported services provided in 2010, this percentage more than doubled in 2011 to 55% of the services provided by the franchises. Asian franchise networks saw the greatest increase in non-family planning services, from just 2% of the services reported for 2010 to 50% in 2011. African social franchising programs initially offered more diversity in its service portfolio than other regions, reporting a higher percentage of non-family planning services in 2010, 56%. Even so, these African networks still continued to diversify in 2011, increasing to 68%. Latin American social franchising programs were almost exclusively focused on family planning in both years. The change in DALYs averted by these services corresponded to the differences shown in the changes in services provided (Table 3). Note that the magnitude of change varies because each country's burden of disease is factored into the calculation of DALYs averted.

**Table 2:** Change in family planning services versus non-family planning services, 2010-11, by region

Region	2010	2011	% Change
**Asia **(*n *= 13)			
Family planning services	174,552,468	305,929,488	75%
Other services	3,834,281	309,438,553	2676%

**Africa **(*n *= 16)			
Family planning services	36,032,303	74,355,350	106%
Other services	45,335,894	160,960,315	255%

**Latin America **(*n *= 3)			
Family planning services	10,537,434	375,934	-96%
Other services	453	na	-100%

**Total **(N = 32)			
Family planning services	221,122,205	380,660,772	72%
Other services	49,170,628	470,398,868	857%

**Table 3:** Change in DALYs averted from family planning services versus non-family planning services, 2010-11, by region

Region	2010	2011	% Change
**Asia **(*n *= 13)			
Family planning DALYs averted	709,484	1,005,291	42%
Other services DALYs averted	155,703	4,300,939	2,662%

**Africa **(*n *= 16)			
Family planning DALYs averted	311,289	944,939	204%
Other services DALYs averted	380,487	3,466,940	811%

**Latin America **(*n *= 3)			
Family planning DALYs averted	14,759	3,645	-75%
Other services DALYs averted	11	0	-100%

**Total **(N = 32)			
Family planning DALYs averted	1,035,532	1,953,874	89%
Other services DALYs averted	536,201	7,767,878	1,349%

### Significance of program output and impact changes, 2010-2011

In paired t-tests comparing the output and impact metrics from the 32 social franchise programs that reported sufficient data for both years, we did not detect significant change in the total number of services provided, DALYs averted, or CYPs. While high degrees of year-to-year variation occurred within a small number of programs, the changes showed both increases and decreases, and led to no common difference across programs. Similarly, no common difference was evident in the sum of services provided which we had categorized as family planning and non-family planning.

### Social franchising program impact in program areas by region

Social franchising programs within the Compendia also show wide variation in the number of DALYs averted during this time period, with variation by program area and by region. The aggregate total for each social franchising program differed greatly, ranging from 110 to 3,340,046 DALYs averted in Africa, and from 1,721 and 4,528,341 DALYs averted in Asia. When analyzed by health program area and region, the total number of DALYs averted also spans a range of results as shown in Table 4. While the number of DALYs averted through reproductive health interventions in Asia grew considerably over the two years, the greatest increase in DALYs averted for Asia was through interventions that target diarrhea. No network in Asia reported products and services in this program area in 2010. In 2011, other social franchising networks reported large numbers of DALYs averted due to diarrhea treatment services. In 2011, one network in Asia generated nearly 4 million DALYs averted from its distribution of over 300 million ORS sachets while another delivered over 4 million diarrhea services (4,042,613 ORS sachets), yielding 47,913 DALYs averted.

In Africa, the program area of malaria experienced the most dramatic increases in DALYs averted. Over the two-year period, six different franchises reported services targeting malaria, with the number of DALYs averted increasing from 145,453 in 2010 to 3,082,540 in 2011. The efforts of a single network accounted for this considerable increase. This social franchise distributed over 9 million (9,053,386) long-lasting, insecticide-treated bednets in 2011, averting 3 million DALYs over the life of the nets.

**Table 4:** DALYs averted from social franchise program service delivery, 2010-11, by program area and region

Program Area	DALYs Averted by Asian Programs		DALYs Averted by African Programs		DALYs Averted by Latin American Programs		DALYs Averted by All Franchises	
	
	2010 (*n *= 13)	2011 (*n *= 13)	2010 (*n *= 16)	2011 (*n *= 16)	2010 (*n *= 3)	2011 (*n *= 4)	2010 (N = 32)	2011 (N = 32)
Family Planning	709,484	1,005,291	311,289	944,939	14,759	3,645	1,035,532	1,953,874

Sexual and Reproductive Health	38,161	32,865	66,354	184,235	11		104,527	217,100

Maternal and Child Health		76,551						76,551

HIV	131	232	128,700	121,977			128,831	122,209

Malaria	38,955	38,872	145,453	3,082,540			184,408	3,121,411

Diarrhea	2,440	4,066,699	36,495	25,167			38,935	4,091,865

Acute Respiratory Illness	8,340	13,820		52,341			8,340	66,160

Tuberculosis	67,675	71,900	3,485	681			71,160	72,581

**Total DALYs averted**	**865,187**	**5,306,229**	**691,776**	**4,411,878**	**14,770**	**3,645**	**1,571,733**	**9,721,752**

### Program reporting consistency

The ratio of the reported number of services provided to the reported number of patient visits for each year offers a data quality check on franchise reporting. While services provided were significantly correlated with the number of outlets in 2010 (r = .418, p < .05) but not in 2011, these data showed a significant correlation with patient volume in both 2010 and 2011 (r = .696, p < .05; r = .383, p < .05, respectively). The variation in the ratio of services provided to patient volume was seen across all three regions and even across years for single franchises. We analyzed 64 pairs of services provided and patients visits. We derived this number by taking each annual report from the franchises separately and removing one annual report from a franchise that failed to report patient visits for a single year. Forty-one annual franchise reports included less than ten services provided per patient visit, predominately less than two. Thirteen annual reports included more than 100 services provided per patient visit, up to 16,632 for one franchise. Among the franchises that reported ratios greater than 100:1, only two reported such high ratios for both years. Note that program affiliation did not determine the high variation, as it occurred among both PSI and non-PSI social franchises.

## Discussion

This evaluation sought to demonstrate the application of using DALYs averted as a standard health impact metric for social franchising. Our results show it is possible to apply a metric based on the DALY to service delivery data from multiple social franchising programs, whether DALYs averted is a new metric for these programs or not. As calculation of the DALYs averted metric just requires social franchise service delivery data as well as sufficient data on disease burden for a country, the PSI DALYs averted models could be applied to non-PSI social franchise data. In some cases, we could not estimate DALYs averted because we did not have sufficient information reported on a franchise program's interventions, such as the number of services provided or what specific disease was targeted. In other cases, we lacked country-specific data, such as disease prevalence, and also could not estimate DALYs averted. However, most social franchises in the Social Franchising Compendia appear to report sufficient detail in service delivery data, and operate in countries in which disease burden data are available. Therefore, as demonstrated, the conversion of this service delivery data into DALYs averted is feasible for global social franchising programs.

Our findings also support the theoretical basis for adopting DALYs averted as a summary measure of program health impact: this new metric can be applied to project health impact across a range of products and services. While patient volume and number of outlets are important process metrics for tracking program progress, they do not indicate health impact. The CYP metric, while commonly understood as a comprehensive measure of program impact unlike these process measures, is actually based on intervention protection and is not context-specific in terms of pregnancy risk in a population. Moreover, the CYP's measurement is limited to family planning services. As demonstrated in this study's analysis of DALYs averted by service type and program area, many social franchising programs offer a diverse mix of services that cannot be measured by CYPs. Certainly, large numbers of DALYs averted from 2010-11 can be attributed to family planning (likely reflecting the reproductive health origins of many social franchising programs). However, treatment and services for health conditions not incorporated in CYPs - but with high importance to current burden of disease [18] and offered by a growing number of social franchise programs - are important drivers of DALYs averted. DALYs averted, therefore, enables social franchisors to capture a diverse array of health services when estimating health impact.

On account of this new metric's wide applicability, the importance of DALYs averted as a summary measure of health impact may be most notable for social franchising programs in which non-family planning services contribute significantly to overall treatment numbers. For programs where family planning is the focus of all or nearly all of the services provided, a shift to DALYs averted is likely to offer only limited improvement in measurement of impact, resulting from the country context adjustments in the family planning DALYs averted model. However, given the current trend in LMICs to broaden clinic offerings beyond specialized, family planning-only services to more integrated and comprehensive care, the benefit of using DALYs averted as the standard health impact metric for social franchising is likely to grow in the future.

In addition to capturing the impact of clinics offering diversified services, the adoption of DALYs averted offers benefits for social franchisors and their stakeholders in programmatic decision making. When tracking ongoing progress and the overall impact of service delivery, social franchisors can calculate, and then compare, DALYs averted at different levels of the network structure. For example, they can compare impact between individual health services, or sum up the DALYs averted from all services to compare the performance of individual clinics within the network. The aggregate health impact estimate also enables benchmarking of an entire franchise network's performance between programs, countries, and regions, comparisons that are currently only possible in social franchising for family planning impact. As the DALYs averted metric incorporates disease burden - and thus, population need - into its impact estimates, social franchisors can use DALYs averted data to help determine which services to emphasize in a particular catchment area, country, or region. While service delivery data can show demand, the potential number of DALYs averted is a better indicator of health needs, and ultimately, health impact.

The DALYs averted metric can also be interpreted alongside other data to reflect program aims and values. The cost per DALY averted is a common indicator of cost-effectiveness. With cost data from a franchising network or for outlets within a franchise, franchise managers can drive toward increased efficiency. A franchise-specific net cost per DALY averted can help social franchisors and stakeholders better consider the opportunities and trade-offs of specific service delivery interventions, leading to greater optimization of care.

### Limitations

Deriving DALYs averted relies on complete and accurate service delivery data, the key limitation of this study. We found evidence of inconsistent service outcome reporting in approximately 10% of programs. Certainly, errors in program reporting are likely present in all health impact measures, making cross-program comparisons inherently challenging. We believe these comparisons are nevertheless still worth attempting. This analysis, and our past experience with many programs, gives us no reason to believe there is systematic and intentional misrepresentation of service delivery information. Rather, as the Social Franchising Compendia aims to include all clinic services provided by its member networks, reporting variations are likely due to poor specification of what data need to be collected.

One implication of the variability identified, however, is that the changes identified through our analysis of 2010-2011 data in DALYs averted and patients seen should be interpreted as indicative rather than absolute. The variability seen here is indicative of changes in the application of franchising as a delivery platform worldwide, rather than changes within specific programs.

Likewise, confusion about the distinction between those health services provided through social franchising versus social marketing distribution channels also persists. The services provided by social franchising programs are clinic-based and include clinical interventions, counseling, and commodity sales. Many clinical interventions, such as IUD insertions, involve one service provided during a single patient visit. Many commodity sales (condoms, cycles of contraceptive pills, or others) could involve large numbers of 'services' provided during a single patient visit. Social marketing is based on commodities only. While social marketing interventions will often involve the same commodities as those in social franchising, these products are usually delivered through non-clinic retail outlets and do not involve patient visits. Despite differences in delivery modality, the two intervention approaches use related market-based models, leading many implementing organizations to operate both, often with the two delivery systems sharing the same overall brand of commodities (e.g., PSI's *PUR *water treatment product to prevent diarrhea).

As a result, confusion in reporting is likely to occur, as suggested by the data used in this study. One notable example of these reporting errors can be seen in some outlier data on commodity sales. In both 2010 and 2011, some social franchising programs stand out as having exceptionally high rates of commodity sales - primarily anti-malarial bednets (LLINs), ORS, and condoms - resulting in some of the highest DALYs averted numbers from individual services in our study, as well as year-on-year volatility in the data. For example, one program in Africa serving approximately 700,000 patients reported the distribution of over 9 million LLINs in a single year. Another program, treating 1.27 million patients in Asia, noted that it delivered over 300 million ORS sachets one year, over 250 sachets per patient. Such high figures suggest that a number of programs are erroneously including non-clinic retail outlet social marketing distribution figures in their social franchising reporting, as we know was the case for a PSI Guatemala program in 2010 that erroneously included non-clinic condom sales in its service delivery data. Moreover, this high year-to-year variation in commodity sales occurs while the number of patient visits remains stable. The high ratios of services to patient volume, particularly those programs with 50:1 or even 16,000:1 ratios, are likely due to an amalgamation of social marketing and social franchising outputs when reporting. Clearer guidelines on the collection and reporting of service delivery data, including how to distinguish between commodities delivered via social franchising versus social marketing channels, are needed to minimize reporting errors in the future. Follow up with outlier programs will also be beneficial.

Another limitation of our analysis is that a number of medical services remain uncounted in our DALYs averted metric, notably antenatal care and safe deliveries. This omission under-credits social franchising programs delivering these services in terms of health impact. If DALYs averted is to become more widely used in social franchising, new DALYs averted models must be developed for these services.

This study is also limited by the models themselves as well as the data fed into them. Determining the number of DALYs averted by an intervention is difficult, requiring complex modeling of the specific burden of disease in each country, and of the effectiveness and health outcomes of each intervention. To create these models, accurate treatment data and population information are essential, yet sometimes challenging to obtain in the LMIC settings where this metric is applied. PSI needs to use proxies and best estimates for some model parameters such as treatment use and wastage, as well as extrapolate population data from nearby countries when local data are unavailable. In addition, development and maintenance of the models has required a considerable investment by PSI. Moving to a broader set of interventions and pressure to develop new models has sometimes compromised the maintenance of models that need to be updated continuously.

## Conclusion

Despite more than a decade of the global health community using DALYs as a measurement of disease burden, the application of this metric for projecting health program impact is still in its nascent stages. For the rapidly emerging social franchise service delivery model, our analysis finds a strong theoretical basis for adopting DALYs averted as its program impact metric. DALYs averted is likely to be the most inclusive and comprehensive measurement available, enabling inter- and intra-program comparisons for tracking program progress and guiding strategic planning and program design. In theory as well as in practice, DALYs averted captures the impact of a wide range of health care services, not limited to the delivery of family planning methods, and thus best represents the breadth of care and treatment offered by a majority of social franchise programs. Our analysis supports this conclusion, even while we found that the application of the DALYs averted remains problematic given current challenges in data reporting and the burden of expanding model development.

The social franchising community of practice offers a useful forum to pilot the DALYs averted metric as a standardized measure for service delivery programs managed by multiple implementing organizations. First, the diversity of health services offered by social franchisors enables opportunities for learning how to best measure program impact in different program areas. Second, the established platform for collaboration and transparent data sharing provides a forum, such as that found in the Social Franchising Compendia, for improving and streamlining data management systems. Certainly, considerable work still needs to be done to assure that service delivery data collected by programs are based on shared parameters and are not subject to the vagaries of interpretation during data entry as appears to have occurred in 2010 and 2011. Motivated by shared interests and the attraction of collaborative work, members of the social franchising community are currently collaborating on developing standards to address these reporting issues. Once this process is accomplished, we expect the social franchising community of practice to be well-positioned to begin using, and eventually standardizing, DALYs averted as its metric for estimating programmatic health impact in all service areas.

## List of abbreviations

DALYs: disability-adjusted life-years; CYPs: couple-years of protection; LMICs: low- and middle-income countries; NGO: non-governmental organization; TB: tuberculosis; HIV/AIDS: human immunodeficiency virus/acute immune deficiency syndrome; PSI: Population Services International; MSI: Marie Stopes International; UCSF: University of California - San Francisco; WHO: World Health Organization; IUD: intrauterine device; ORS: oral rehydration salts; LLINs: long-lasting, insecticide-treated nets; PDF: Portable Document Format; USAID: United States Agency for International Development; LAM: Lactation Amenorrheal Method; CAC: comprehensive abortion care; MVA: manual vacuum aspiration; CDK: clean delivery kit; PMTCT: prevention of mother-to-child transmission of HIV; ITN: insecticide-treated net; RDK: rapid diagnostic kit; RDT: rapid diagnostic testing; ACT: artemisinin-based combination therapy; GDP: gross domestic product; HCT: HIV counseling and testing

## Competing interests

The authors declare that they have no competing interests.

## Authors' contributions

DM conceived of the analysis and wrote the first draft. LN led the adaptation of the DALYs averted models to the social franchising data and conducted the primary analysis. SD reviewed findings, advised on interpretation, and led the rewriting of the second draft. AR conducted the sub-analysis. AR and LN wrote the findings. All authors contributed equally to the background, interpretation, and discussion. All authors read and approved the final manuscript.

## Supplementary Material

Additional file 1 **Description of all social franchising networks in Social Franchising Compendia, 2011**. This table provides a profile of each social franchising network that is a member of the Social Franchising Compendia. Click here for file

Additional file 2 **Reported services corresponding to program areas from all social franchising networks**. This table lists each of the health services reported by all of the social franchising networks, arranged by program area. Click here for file

Additional file 3 **Change in program output and impact data, 2010-11**. This file shows the change in program outputs and impacts from 2010 to 2011 among the 32 social franchising programs reporting data for both years of interest. Click here for file

## Declarations

This article is part of the supplement of *BMC Public Health *Volume 13, Supplement 2, 2013: Use of health impact metrics for programmatic decision making in global health. Population Services International, a registered non-profit organization, provided the funding for the publication of this supplement. The full contents of the supplement are available online at http://www.biomedcentral.com/bmcpublichealth/supplements/13/S2.
