# Supplementary material for: Applying the disability-adjusted life year to track health impact of social franchise programs in low- and middle-income countries
Source: BMC Public Health. 2013 Jun 17;13(Suppl 2):S4. doi: 10.1186/1471-2458-13-S2-S4 (PMC3684545; doi:10.1186/1471-2458-13-S2-S4)
Supplement: Additional file 1 — Description of all social franchising networks in Social Franchising Compendia, 2011. This table provides a profile of each social franchising network that is a member of the Social Franchising Compendia. [file 1471-2458-13-S2-S4-S1.PDF]

Additional file 1. Description of all social franchising networks in Social Franchising Compendia, 2011

Region	Country	Franchise	Affiliation	Program Launch Year	Number of Outlets	Patient Volume (# of patient visits)	Program Area+								
							FP	SRH	MCH	HIV	Mal	Dia	ARI	TB	Other
Asia	Bangladesh	Smiling Sun^	Other	2008	9,144	25,324,740	Δ	Δ	Δ		Δ	Δ	Δ	Δ	Δ
Asia	Bangladesh	Social Marketing Company (Blue Star Bangladesh)	Other	1998	4,000	1,269,130	Δ	Δ	Δ			Δ		Δ	
Asia	Cambodia	Sun Quality Health Network	PSI	2002	213	27,430	Δ	Δ							
Asia	India	Drishtee Health Franchisee^**	Other	2007	50	9,500			Δ						
Asia	India	Franchise Surya Clinic	DKT	1998	105	6,812	Δ	Δ							
Asia	India	MerryGold Health Network	Other	2007	11,292	536,680	Δ	Δ	Δ	Δ					
Asia	India	Saadhan Network	PSI	2008	880	54,520	Δ	Δ							
Asia	India	SKY Health Network	Other	2008	5,980	45,644	Δ	Δ	Δ				Δ	Δ	Δ
Asia	Indonesia	Andalan Network of Clinics	DKT	2008	2,229	10,000	Δ								
Asia	Lao PDR	Sun Quality Health Network	PSI	2011	55	721								Δ	
Asia	Myanmar	Sun Quality Health and Sun Primary Health	PSI	2001	1,498	2,120,000	Δ			Δ	Δ	Δ	Δ	Δ	
Asia	Nepal	Mahila Swastha Sewa	PSI	2010	408	49,816	Δ	Δ				Δ			
Asia	Nepal	Sangini Franchising	Other	1994	3,365	81,900	Δ	Δ				Δ			
Asia	Pakistan	Sabz Sitara (Greenstar)	PSI	1995	7,289	3,818,880	Δ	Δ	Δ					Δ	
Asia	Pakistan	Suraj	MSI	2008	190	133,843	Δ								
Asia	Philippines	BlueStar Pilipinas	Other	2008	266	150,585	Δ	Δ							
Asia	Philippines	POPSHOP	DKT	2005	300	100,000	Δ			Δ					
Asia	Philippines	Well-Family Midwife Clinic^	Other	1997	132	23,760	Δ	Δ	Δ	Δ	Δ	Δ	Δ		Δ
Asia	Vietnam	BlueStar Vietnam	MSI	2008	300	994,270	Δ	Δ							
Asia	Vietnam	tinh chi em	MSI	2007	216	887,412	Δ	Δ							
Africa	Benin	Protection de la Famille (ProFam)	PSI	2004	152	50,000	Δ			Δ	Δ	Δ			
Africa	Burundi	LifeNet International**	Other	2008	10	NA					Δ				Δ
Africa	Cameroon	ProFam	PSI	2003	71	6,843	Δ	Δ	Δ	Δ	Δ		Δ	Δ	
Africa	Congo-Kinshasa	CONFIANCE NETWORK	PSI	2003	133	718,351	Δ	Δ		Δ	Δ	Δ	Δ		

Region	Country	Franchise	Affiliation	Program Launch Year	Number of Outlets	Patient Volume (# of patient visits)	Program Area+								
							FP	SRH	MCH	HIV	Mal	Dia	ARI	TB	Other
Africa	Ethiopia	BlueStar Ethiopia Social Franchise Network	MSI	2007	356	258,957	Δ	Δ							
Africa	Ghana	BlueStar	MSI	2008	113	152,484	Δ	Δ							
Africa	Ghana	HEALTHKEEPERS NETWORK	Other	2006		520,000	Δ			Δ					
Africa	Ghana	MicroClinic International^**	Other	2009	3	47,890									
Africa	Kenya	AMUA	MSI	2004	250	402,184	Δ	Δ		Δ					
Africa	Kenya	Gold Star Network	Other	2006	192	1,900	Δ			Δ				Δ	
Africa	Kenya	KMET PACNET^**	Other	1995	350	15,000									
Africa	Kenya	Tunza Family Health Network	PSI	2008	258	440,617	Δ	Δ		Δ					
Africa	Kenya, Rwanda	Child and Family Wellness (CFW)	Other	2000	71	400,000	Δ				Δ	Δ			Δ
Africa	Madagascar	BlueStar Healthcare Network	MSI	2010	133	75,600	Δ	Δ							
Africa	Madagascar	PROFEMINA^	PSI	2009	98	16,461	Δ								
Africa	Madagascar	TOP Réseau	PSI	2001	193	167,441	Δ	Δ		Δ					
Africa	Malawi	BlueStar Healthcare Network	MSI	2008	34	27,813	Δ			Δ					
Africa	Mali	ProFam	PSI	2005	66	7,638	Δ			Δ					
Africa	Mozamibique	Intimo	DKT	2011	11	3,100	Δ								
Africa	Nigeria	Happy Mothers Network	PSI	2010	290	19,363	Δ	Δ							
Africa	Nigeria	Hygeia Community Health Plan	Other	2006	22	95,218				Δ	Δ			Δ	
Africa	Sierra Leone	BlueStar Sierra Leone	MSI	2008	132	66,408	Δ	Δ							
Africa	Somaliland	BulshoKaab**	PSI	2011	50										
Africa	South Africa	BlueStar South Africa^	MSI	2010	20	37	Δ	Δ							
Africa	South Africa	New Start	PSI	2002	12	147,135				Δ					
Africa	South Africa	Public-Private Partnerships in ART Patient Management	Other	2005	38	2,430				Δ				Δ	
Africa	Sudan	Mother Health	DKT	2011	25	35,000									
Africa	Tanzania	Familia	PSI	2009	255	30,000	Δ								
Africa	Togo	POMEFA	PSI	2009	79	60,000	Δ								
Africa	Uganda	Living Goods**	Other	2007		436,296									

Region	Country	Franchise	Affiliation	Program Launch Year	Number of Outlets	Patient Volume (# of patient visits)	Program Area+								
							FP	SRH	MCH	HIV	Mal	Dia	ARI	TB	Other
Africa	Uganda	ProFam	PSI	2008	119	29,537	Δ	Δ		Δ	Δ	Δ			
Africa	Zambia	NewStart	PSI	2002	8	65,662				Δ					
Africa	Zimbabwe	New Life	PSI	2003	28	152,000	Δ							Δ	
Africa	Zimbabwe	New Start**	PSI	1999	40	380,000	Δ			Δ				Δ	
Latin America	Guatemala	Red Segura	PSI	2010	214	6,876	Δ								
Latin America	El Salvador	Red Segura	PSI	2009	22	883	Δ								
Latin America	Nicaragua	Red Segura	PSI	2011	52	26,267	Δ								
Latin America	Peru	RedPlan Salud	Other	2002	1,723	632,895	Δ	Δ		Δ					

*2010 data are used for certain programs in which 2011 data were not available
^2010 data
**Excluded from study sample
+ Program Area:
FP – Family planning
SRH – Sexual and reproductive health
MCH – Maternal and child health (other than diarrhea and ARI)
HIV – HIV/AIDS
Mal – Malaria
TB – Tuberculosis
Dia – Diarrhea
ARI – Acute Respiratory Illness
Other - Other
