# Supplementary material for: Applying the disability-adjusted life year to track health impact of social franchise programs in low- and middle-income countries
Source: BMC Public Health. 2013 Jun 17;13(Suppl 2):S4. doi: 10.1186/1471-2458-13-S2-S4 (PMC3684545; doi:10.1186/1471-2458-13-S2-S4)
Supplement: Additional file 2 — Reported services corresponding to program areas from all social franchising networks. This table lists each of the health services reported by all of the social franchising networks, arranged by program area. [file 1471-2458-13-S2-S4-S2.PDF]

Additional file 2. Reported services corresponding to program areas from all social franchising networks

Program Area	Product/Service
**Family Planning**	
	3-year implant
	4-year implant
	5 year IUD (e.g. LNG-IUS) (# of inserted IUDs)
	5-year implant
	Copper-T 380-A IUD (# of inserted IUDs)
	Cyclofem monthly injectable (or other monthly injectable) (# of doses)
	Depo-Provera injectable (or other 3-month injectable) (# of doses)
	Emergency contraception
	Female condom
	Fertility awareness methods (# of trained adopters)
	IUD (# of inserted IUDs)
	Lactational amenorrhea method (LAM) (# of active users)
	Male condom
	Noristerat injectable (or other 2-month injectable) (# of doses)
	Oral contraceptives
	Standard days method (# of trained adopters)
	Sterilization (male and female)
	Vaginal foaming tablets (# of units)
	Newlywed counseling
**Sexual and Reproductive Health (non-family planning)**	
	Cervical cancer screening and referral
	Medical abortion (misprostol/Cytotec)
	Misoprostol
	Post abortion care: sublingual misoprostol (# of tablets)
	Surgical abortion (CAC, menstrual regulation, MVA)
	Post-abortion care (not specified)
	Antenatal care
	Clean delivery kits (CDKs)
	Emergency obstetric care
	Labor and delivery
	Labor and delivery/emergency obstetric care
	Postnatal care
**Maternal and Child Health**	
	Baby Active
	Micronutrient powder (# sachets)
	Vaccinations (# children receiving any vaccinations)
	Pediatric consultations
**HIV**	
	Antiretroviral therapy (tablets dispensed)
	Lubricants
	Male circumcision
	Opportunistic infection management (# individuals treated)
	PMTCT (Nevirapine/NVP)(dose for mother and baby)
	PMTCT (Nevirapine/NVP)(single dose for mother)
	HIV testing and counseling

Program Area	Product/Service
**Malaria**	
	Insecticide-treated nets (ITNs)
	Long-lasting, insecticide-treated nets (LLINs)
	Malaria testing (Rapid Diagnostic Kit/RDK, RDT)
	Malaria rapid diagnostic test with positive result
	Treatment (ACT)
	Treatment (Non-ACT)
**Diarrhea**	
	Diarrheal disease treatment
	ORS
	Water purification treatment
	Zinc (tablet)
**Acute Respiratory Infection**	
	Pneumonia (testing)
	Pneumonia (treatment)
**Tuberculosis**	
	TB cases diagnosed/detected
	TB cases initiating treatment
	TB cases that completed treatment
	TB referrals for sputum test
**Other**	
	Laboratory services
	General medicine
	Vision screening and glasses provision
	Vitamins (# pills)
