# Supplementary material for: Applying the disability-adjusted life year to track health impact of social franchise programs in low- and middle-income countries
Source: BMC Public Health. 2013 Jun 17;13(Suppl 2):S4. doi: 10.1186/1471-2458-13-S2-S4 (PMC3684545; doi:10.1186/1471-2458-13-S2-S4)
Supplement: Additional file 3 — Change in program output and impact data, 2010-11. This file shows the change in program outputs and impacts from 2010 to 2011 among the 32 social franchising programs reporting data for both years of interest. [file 1471-2458-13-S2-S4-S3.PDF]

Additional file 3. Change in program output and impact data, 2010-11

Country	Franchise	Services provided*			DALYs averted			CYPs			Number of outlets			Patient volume (# of patient visits)		
		2010	2011	% change	2010	2011	% change	2010	2011	% change	2010	2011	% change	2010	2011	% change
Bangladesh	Social Marketing Company (Blue Star Bangladesh)	6,918,433	477,280,734	6,799%	57,962	4,528,341	7,713%	461,813	3,722,833	706%	3,336	4,000	20%	1,202,785	1,269,130	6%
India	Franchise Surya Clinic	33,446,840	7,513	-100%	93,679	5,968	-94%	672,754	82,085	-88%	105	105	0%	2,011	6,812	239%
India	MerryGold Health Network	28,913	336,052	1,062%	13,531	8,710	-36%	157,749	107,451	-32%	10,426	11,292	8%	414,000	536,680	30%
India	Saadhan Network	278,514	195,121	-30%	52,632	30,454	-42%	393,113	179,956	-54%	4,235	880	-79%	49,044	54,520	11%
India	SKY Health Network	1,698,995	375,797	-78%	6,139	7,470	22%	45,418	67,474	49%	2,726	5,980	119%	41,470	45,644	10%
Myanmar	Sun Quality Health and Sun Primary Health	2,074,309	2,653,411	28%	120,230	149,613	24%	263,740	280,500	6%	2,254	1,498	-34%	1,812,109	2,120,000	17%
Nepal	Mahila Swastha Sewa	649,700	24,039	-96%	18,772	7,686	-59%	132,760	86,429	-35%	300	408	36%	27,000	49,816	85%
Nepal	Sangini Franchising	17,388,786	19,004,800	9%	57,162	100,836	76%	364,052	346,777	-5%	3,008	3,365	12%		81,900	NA
Pakistan	Sabz Sitara (Greenstar)	115,166,643	114,196,470	-1%	401,254	389,480	-3%	2,821,491	2,072,616	-27%	8,000	7,289	-9%	3,000,000	3,818,880	27%
Pakistan	Suraj	200,807	266,590	33%	24,987	34,066	36%	192,485	280,162	46%	100	190	90%	98,370	133,843	36%
Philippines	BlueStar Pilipinas	179,835	191,735	7%	8,506	11,200	32%	139,588	238,611	71%	307	266	-13%	135,696	150,585	11%
Vietnam	BlueStar Vietnam	198,353	444,909	124%	8,611	27,396	218%	64,003	374,889	486%	223	300	35%	391,515	994,270	154%
Vietnam	tinh chi em	156,621	390,870	150%	1,721	5,007	191%	40,281	94,746	135%	186	216	16%	431,797	887,412	106%
Benin	Protection de la Famille (ProFam)	17,153,021	23,071,797	35%	189,316	126,357	-33%	141,889	133,636	-6%	50	152	204%	9,178	50,000	445%
Cameroon	ProFam	17,217	22,078,826	128,139%	3,904	122,851	3,047%	27,404	219,253	700%	24	71	196%	11,167	6,843	-39%
Congo-Kinshasa	CONFIANCE NETWORK	1,011,549	181,921,237	17,884%	31,751	3,340,046	10,420%	130,870	487,154	272%	115	133	16%	596,785	718,351	20%
Ghana	BlueStar	108,890	144,731	33%	2,583	9,358	262%	10,749	19,204	79%	112	113	1%	111,888	152,484	36%

Country	Franchise	Services provided*			DALYs averted			CYPs			Number of outlets			Patient volume (# of patient visits)		
		2010	2011	% change	2010	2011	% change	2010	2011	% change	2010	2011	% change	2010	2011	% change
Kenya	Gold Star Network	1,454,270	1,570,049	8%	4,609	7,526	63%	3,083	9,152	197%	526	192	-63%	116,636	1,900	-98%
Kenya	Tunza Family Health Network	19,485	382,853	1,865%	10,682	23,912	124%	69,668	152,457	119%	257	258	0%	162,431	440,617	171%
Madagascar	BlueStar Healthcare Network	47,103	3,228,595	6,754%	3,364	603,105	17,829%	21,798	3,299,693	15,037%	104	133	28%	45,869	75,600	65%
Madagascar	TOP Réseau	284,220	242,107	-15%	1,881	10,568	462%	7,947	81,514	926%	140	193	38%	129,114	167,441	30%
Malawi	BlueStar Healthcare Network	19,286	28,147	46%	2,436	3,707	52%	11,181	13,671	22%	49	34	-31%	20,825	27,813	34%
Mali	ProFam	4,723	7,416	57%	567	1,897	234%	2,870	10,888	279%	54	66	22%	4,906	7,638	56%
Nigeria	Happy Mothers Network	238,188	19,363	-92%	53,334	13,203	-75%	69,600	87,274	25%	150	290	93%	20,000	19,363	-3%
Nigeria	Hygeia Community Health Plan	14,720,095	59,836	-100%	46,521	6,579	-86%			NA	25	22	-12%	66,526	95,218	43%
South Africa	Public-Private Partnerships in ART Patient Management	1,095,070	1,774,116	62%	2,859	4,811	68%			NA	19	38	100%	1,456	2,430	67%
Togo	POMEFA	1,015	55,772	5,395%	110	4,480	3,967%	874	38,718	4,328%	9	79	778%	1,038	60,000	5,680%
Uganda	ProFam	236,865	222,577	-6%	40,349	23,861	-41%	66,996	129,732	94%	145	119	-18%	20,912	29,537	41%
Zimbabwe	New Start	44,957,200	508,243	-99%	297,509	109,618	-63%	327,133	10,983	-97%	41	40	-2%	380,000	380,000	0%
Guatemala	Red Segura	10,345,281	39,241	-100%	12,818	1,445	-89%	135,635	33,351	-75%	111	214	93%	4,580	6,876	50%
El Salvador	Red Segura	6,713	27,337	307%	729	508	-30%	23,571	20,731	-12%	50	22	-56%	3,102	883	-72%
Peru	RedPlan Salud	185,893	309,356	66%	1,223	1,692	38%	24,434	36,772	50%	1,657	1,723	4%	618,596	632,895	2%

*For reported services that correspond to DALYs averted and CYP coefficients
